# Expression and Clinical Significance of Matrix Metalloproteinase-9 in Lymphatic Invasiveness and Metastasis of Breast Cancer

**DOI:** 10.1371/journal.pone.0097804

**Published:** 2014-05-20

**Authors:** Qiu-Wan Wu, Qing-Mo Yang, Yu-Fan Huang, Hong-Qiang She, Jing Liang, Qiao-Lu Yang, Zhi-Ming Zhang

**Affiliations:** Xiamen Cancer Center and Department of Breast Surgery, The First Affiliated Hospital of Xiamen University, Xiamen, China; University of North Carolina School of Medicine, United States of America

## Abstract

**Background:**

Matrix metalloproteinase 9 (MMP-9) is a type-IV collagenase that is highly expressed in breast cancer, but its exact role in tumor progression and metastasis is unclear.

**Methods:**

*MMP-9* mRNA and protein expression was examined by real-time reverse transcriptase PCR and immunohistochemical staining, respectively, in 41 breast cancer specimens with matched peritumoral benign breast epithelial tissue and suspicious metastatic axillary lymph nodes. Lymph vessels were labeled with D2-40 and lymphatic microvessel density (LMVD) was calculated. Correlation of MMP-9 protein expression with clinicopathological parameters and LMVD was also evaluated.

**Results:**

MMP-9^+^ staining in breast cancer specimens (35/41, 85.4%) was higher than in matched epithelium (21/41, 51.2%; *P*<0.05) and lymph nodes (13/41, 31.7%; *P*<0.001). Higher *MMP-9* mRNA expression was also detected in tumor specimens compared with matched epithelial tissues and lymph nodes (*P*<0.05). Elevated MMP-9 expression was correlated with lymph node metastasis and LMVD (*P*<0.05).

**Conclusion:**

MMP-9 was overexpressed in breast cancer specimens compared with peritumoral benign breast epithelium and lymph nodes. Moreover, its expression in the matched epithelium and lymph nodes was positively associated with lymph node metastasis, and its expression in lymph nodes was positively associated with lymphangiogenesis in breast cancer. Thus, MMP-9 is a potential marker for breast cancer progression.

## Introduction

Breast cancer is the most common tumor among women[1]and often metastasizes to the axillary lymph nodes. The lymphatic system has a complex role in metastasis[2],both as a conduit for tumor cells and as a source of immunocytes to create immune barriers. Increased numbers of lymphatic microvessels reflect lymphangiogenesis and lymphatic vessel remodeling to provide metastasis dissemination paths, and also predicts poor prognosis[3].

Matrix metalloproteinase-9 (MMP-9), also known as gelatinase B, is a 92-kDa zinc-dependent endopeptidase that promotes degradation of type IV collagen, the main component of basement membrane[4].Interestingly, although MMP-9 is highly expressed in breast cancer, its expression is rarely associated with malignant factors[5], [6].Furthermore, conflicting studies using experimental tumor systems reported correlations or lack thereof of increased MMP-9 expression with metastasis[7].

We undertook the present study to gain further insight into the expression pattern of MMP-9 in invasive breast cancer and its relationship with clinicopathological characteristics, lymph vessel density (LMVD), tumor metastasis potential and estrogen receptor (ER), progesterone receptor (PR), and HER2/neu status.

## Methods

### Patients and specimens

This work was performed in compliance with the Helsinki Declaration. Approval to conduct this study was obtained from the Human Subjects Office of the Institutional Research Board at Xiamen University before the project was undertaken. Written informed consent was obtained from all patients or their relatives.

Fresh surgical specimens were obtained from 41 randomly selected female patients who had undergone modified radical mastectomy in the Department of Breast Surgery of the First Affiliated Hospital of Xiamen University, from September 2009 to September 2010. The average age at time of diagnosis was 53 years (range, 29–76 years). None of the patients had received preoperative treatment, such as radiotherapy or chemotherapy. Metastatic tumors from other tissue origins were excluded. Three types of specimens were obtained from each patient: cancer tissue, a random suspicious (by naked eye) metastatic ipsilateral axillary lymph node and peritumoral benign breast epithelium located >5 cm from the tumor margins. Each specimen was then divided into two parts: one was snap-frozen in liquid nitrogen for RNA extraction, and the other was fixed for immunostaining and routine pathological examination.

Cases were evaluated for histological type, tumor grade, histological grade (according to Nottingham histological scores)[8], lymph node metastasis, and ER, PR, and HER2/neu status, according to the American Joint Committee on Cancer (AJCC, 7^th^ ed., 2010).

### Real-time RT-PCR

Total RNA was extracted with Trizol reagent (Invitrogen, USA) in accordance with the manufacturer's instructions. Reverse transcription of total RNA to cDNA was carried out using TaKaRa Reverse Transcription Reagents (Takara Bio, Japan) at 37°C for 15 min, and then 85°C for 5 s. Primers were designed using Primer Premier 5.0 software (Premier, Canada) and synthesized by Invitrogen (USA). Sequence-specific primers for *MMP-9* mRNA (GenBank Accession No. NM 004994.2) were as follows: forward (F): 5′-CATTTCGACGATGACGAGTTGT-3′; reverse (R): 5′-CGGGTGTAGAGTCTCTCGC-3′. *GAPDH* mRNA was employed as reference using the following primers: F: 5′-GAAGGTGAAGGTCGGAGTC-3′; R: 5′-GAAGATGGTGATGGGATTTC-3′. Real-time quantitative PCR was performed with the Takara SYBR Premix Ex Taq II PCR kit (Takara Bio) in a Roche Lightcycler 480 (Roche, Switzerland) PCR machine. The reaction was performed in a 10 µl volume using the following protocol: denaturation at 95°C for 5 s, annealing at 58°C for 15 s, and extension at 72°C for 20 s, for 40 cycles. The relative amount of gene expression was calculated using the 2^−ΔCt^ method, which was then expressed by the mean ± standard error of the mean (S.E.M.)[9]. Experiments were performed in triplicate. The mean Ct values were calculated from triplicate PCRs for both *MMP-9* and *GAPDH*, and the ΔCt value for each specimen was obtained by subtracting these two values. Then the relative amount of MMP-9 expression was calculated by 2^−ΔCt^ (Table 1). Finally, the relative amount of MMP-9 expression in breast benign tissue was standardized as 1. The fold change in expression of MMP-9 between the breast cancer tissue (or lymph nodes) and breast benign tissue was the ratio of these two 2^−ΔCt^, that is 2^−ΔΔCt^.

**Table 1:** MMP-9 expression in breast cancer tissues, benign epithelium and lymph nodes.

Tissue type	N	*MMP-9* mRNA	*F*	MMP-9^+^%	MMP-9 stain grades				*Χ* ^2^
					−	+	++	+++	
Cancer tissues	41	1.01±0.14^A^		85.4^D^	6	13	12	10	
Benign tissues	41	0.32±0.17^B^		51.2^E^	20	6	12	3	
Lymph nodes	41	0.51±0.04^C^	14.071***	31.7^F^	28	9	3	1	31.99***

***P<0.05 is considered statistically significant.

A vs. B, A vs. C: *P*<0.05, respectively, and B vs. C. *P*>0.05.

χ^2^ division: α  =  0.05/4  =  0.0125; D vs. E and D vs. F: *P*<0.0125, respectively; E vs. F: *P*>0.0125.

### IHC staining and evaluation

IHC staining was performed using standard methods. Briefly, 4-mm-thick sections of formalin-fixed, paraffin-embedded tissues were deparaffinized and rehydrated stepwise. Samples were microwaved in citrate buffer (pH 6.0) for 1.5 min to quench endogenous peroxidase activity and nonspecific binding was blocked by incubation in 10% non-immune goat serum (Santa Cruz Biotechnology, USA.) for 10 min. Antibodies used were as follows: anti-MMP-9 antibody (1∶50; Abcam, UK), D2-40 (1∶40; Abcam), ER (1: 50; Dako Cytomation, Denmark), PgR (1: 50; Dako Cytomation), and HER2 (1: 1500; Dako Cytomation). Sections were then incubated in primary antibody for 2 h at room temperature. After rinsing, the sections were incubated with EnVision Detection Systems (Dako Cytomation), then counterstained with hematoxylin, dehydrated, and mounted. Positive controls consisted of arrays of different tissues or specimens found to express MMP-9 protein in previous studies. The negative controls were processed using the same procedure, except that 10% nonimmune mouse serum (Santa Cruz Biotechnology.) was used in place of the primary antibody. No detectable staining was observed in all negative control slides. Hematoxylin–eosin-stained slides of all cases were reviewed to confirm their diagnoses and histopathological characteristics. Morphometric analyses were estimated independently by two observers who were blinded to patient clinical characteristics.

LMVD was assessed by counting the number of D2-40 immunostained vessels on tissue sections. As previously reported[10],we first identified the area containing the most stained vessels (‘hot spots’) by scanning the sections at low magnification (40×), and then counted the positive vessels in two high magnification fields (200×). These vessels were defined as lymphatic if they were lined by a single layer of immunopositive flattened endothelial cells with a vascular lumen, with or without the presence of lymphocytes but absent of erythrocytes[11].LMVD in tumor sections included those within the tumor or at the tumor periphery. Mean visual microvessel density was calculated as the average of four counts (two authors and two microscopic fields). Discrepancies between the observers were found in <10% of the slides examined, and a consensus was reached on further review.

The staining intensity of MMP-9 and the number of stained cells were both taken into consideration. Stains were scored on a scale of 0–3. A score of 0 (−) was assigned for no staining or weak staining in <10% positive cells; 1 (+) for weak to moderate staining in 11–20% of cells; 2 (++) for moderate to strong staining in 21–50%; or 3 (+++) for strong staining in >50% of cells. Samples were considered to be MMP-9^+^ when ≥10% of cells were immunoreactive, as previously described[12].Staining for ER, PR and HER2 was evaluated using ASCO guidelines.

### Statistics

Data were analyzed using SPSS (ver. 17.0, Chicago, IL, USA). Data of normal distribution were applied to parametric statistics and expressed as mean ± standard error of the mean (S.E.M.). Unpaired Student's *t*-tests were used to compare two sets of data and one-way analysis of variance (ANOVA) with Dunnett's post-test for comparisons of more than two data sets. Non-parametric statistics (Chi-square test, Yates' correction or Fisher's exact test for qualitative independent variables) were applied to data with abnormal distribution. Bonferroni's correction of the α-value was used for multiple comparisons. Mann–Whitney U tests were applied to nonparametric tests of two independent variables. Kruskal–Wallis H tests were applied to nonparametric tests of multiple independent variables (Table 2). Correlations between two variables were assessed using Spearman's rho test. All statistical tests were two-sided and *P* values of <0.05 were considered statistically significant.

**Table 2:** Correlation of MMP-9 expression with clinicopathological characteristics.

		MMP-9 expression					
Clinicopathological characteristics		Cancer tissues		Benign tissues		Lymph nodes	
	Case	Positive	*P*	Positive	*P*	Positive	*P*
Tumor							
T1	15	12	0.643^b^	7	0.815^b^	4	0.350^b^
T2	23	20		12		9	
T3	3	3		2		0	
Lymph node metastasis							
−	22	20	0.286^a^	6	0.001^a^ *	0	0.000^a^ *
+	19	15		15		13	
ER							
−	10	8	0.392^a^	6	0.528^a^	1	0.094^a^
+, ++, +++	31	27		15		12	
PR							
−	8	6	0.361^a^	4	0.939^a^	1	0.199^a^
+, ++, +++	33	29		17		12	
HER2†							
−	31	26	0.777^a^	13	0.540^a^	9	0.622^a^
+	10	9		8		4	
Histological grade							
I	4	4	0.327^b^	2	0.968^b^	1	0.905^b^
II	26	23		13		8	
III	11	8		6		4	
Histological type							
Duct carcinoma	36	30	0.329^a^	19	0.597^a^	12	0.553^a^
Other	5	5		2		1	

a Mann–Whitney U test;

b Kruskal–Wallis H test.

“+”, “++” and “+++” for MMP-9 immunochemistry staining were grouped together as “+”.

*P<0.05 is considered statistically significant.

† IHC2+ was confirmed by FISH.

## Results

### LMVD assessment

**Figure 1:**
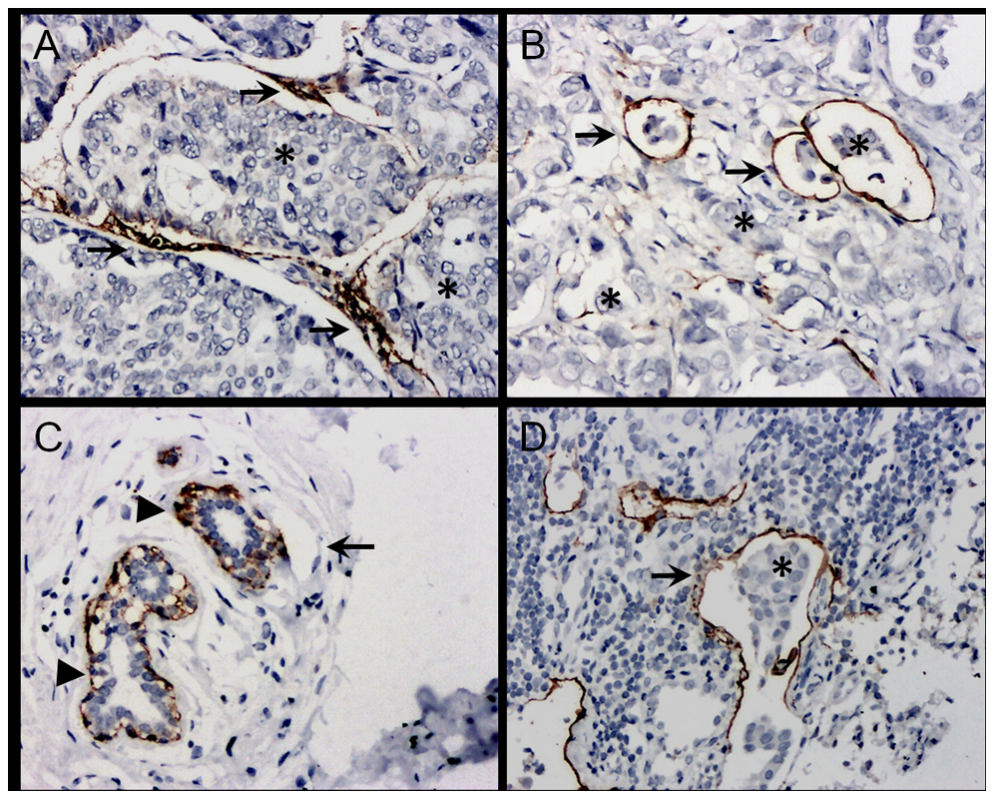
Immunohistochemical staining of D2-40. **A**. Lymph vascular structures in the central tumor were linear, flattened, cluttered and densely arrayed; **B**. Stained lymphatic microvessels (long arrows) in a peripheral tumor with dilated tubes. Tumor cell clusters (indicated by asterisks) were not stained. A cluster of tumor cells in a stained vessel was recognized as lymphatic vessel invasion (LVI); **C**. Benign breast epithelium. Luminal epithelial cells with D2-40^−^ staining. Myoepithelial cells from normal ducts and lobules exhibited D2-40^+^ immunostaining (arrow heads), but with a granular, branching membranous staining pattern distinct from the characteristic staining pattern of lymphatic endothelium shown in B. Capillary vessels were not stained (indicated by long arrow); **D**. Metastatic lymph nodes. Dilated lymphatic microvessel sub-capsular metastasis with no tumor cell staining (indicated by asterisks), defined as ‘tumor emboli’ (indicated by arrow). Magnification, 200×.

D2-40 is a commercially available mouse monoclonal antibody against human podoplanin, which is a mucin-type transmembrane protein in lymphatic endothelial cells[13].The antibody is a highly specific marker for lymphatic endothelium and has proven valuable in distinguishing lymph vessels from blood vessels and in detecting lymphatic invasion in various malignant neoplasms[11], [14], [15].In the present study, D2-40 staining was mainly detected in lymphatic endothelial cells, while tumor cells and blood vessels exhibited no staining (Figure 1B and 1C). Ductal cancer *in situ* (DCIS) foci displayed weaker residual discontinuous myoepithelial staining. Tumor lymphatic vessel invasion (LVI) was established when at least one tumor cell cluster (‘tumor emboli’) was clearly visible inside a D2-40^+^ lymph vessel, in accordance with Hasebe *et al*. (Figure 1B)[16]. Despite evidence of D2-40 staining in the basal epithelial cell layers of the epidermis, the myoepithelial cells of human breast tissue, and prostate and salivary gland tissue, the morphology of these cells differed from that of lymphatic endothelium (Figure 1C)[17], [18].

The average number of LMV per field (200×) in breast cancer specimens was 12.90±1.71 (range, 1.01–42.10). For the matched benign breast tissues and lymph nodes, the LMVDs were 2.14±0.17 (range, 1.03–5.22) and 5.41±0.39 (range, 1.03–32.16) LMV per field (200×), respectively. LMVDs significantly differed among the three tissue types (Friedman test, *P*<0.01). Dunnett's post-test showed that the LMVD in tumor tissues was higher than in breast epithelium or lymph nodes (both *P*<0.01). However, the LMVD did not significantly differ between benign tissues and lymph nodes (*P*>0.05). These data indicate that the LMVD in breast cancer tissue was significantly higher than in matched benign tissue. Lymphatic vessels are known to have discontinuous basement membranes and to lack tight interendothelial junctions, and are therefore believed to be more easily infiltrated by tumor cells[2].Enhanced LMVD provides increased opportunities for tumor cells to invade lymphatic vessels [18].

### MMP-9 expression and clinical characteristics

**Figure 2:**
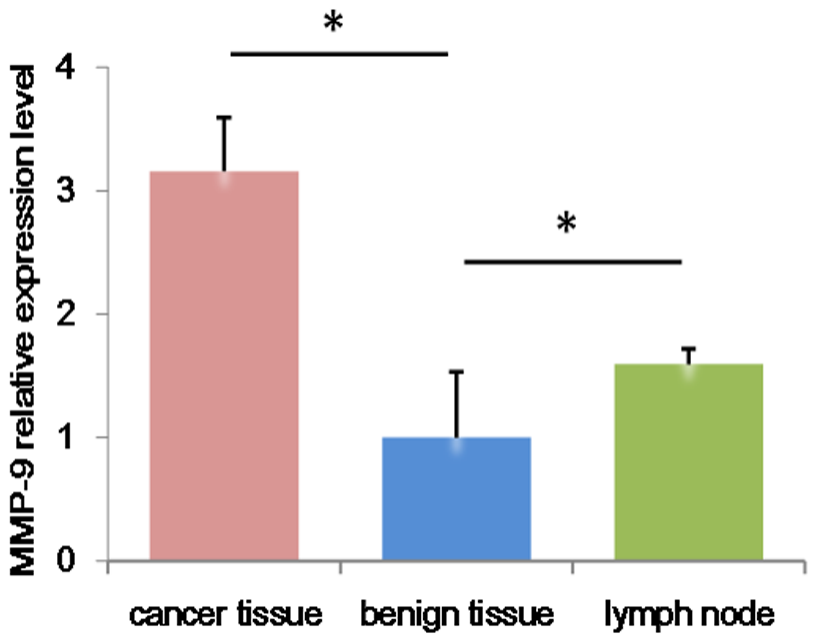
*MMP-9* mRNA expression in breast cancer tissue, breast benign tissue and lymph nodes. Experiments were run in triplicate. *P<0.05 was considered statistically significant.

The relative amount of *MMP-9* mRNA expression differed significantly among the three tissue types (one-way ANOVA, *P*<0.01; Table 1), and the fold change in *MMP-9* mRNA expression was significantly higher in cancer tissue than in benign tissue or lymph node samples (Tukey's test, *P*<0.05; Figure 2).

**Figure 3:**
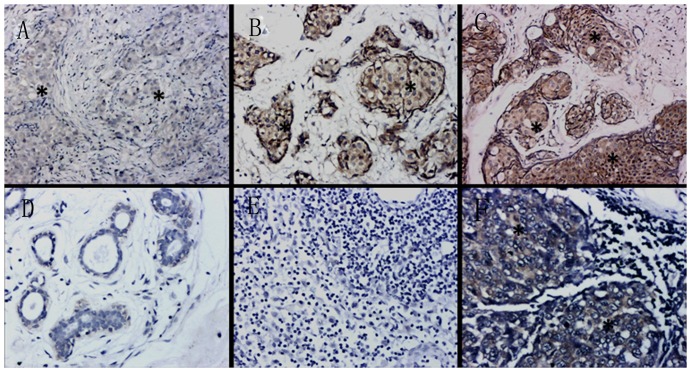
Representative immunohistochemical staining of MMP-9. Breast cancer sections exhibiting MMP-9^-^ staining (**A**), B. MMP-9^++^ staining (**B**), and C. MMP-9^+++^ staining (**C**). MMP-9 was detected primarily the cytoplasm of breast cancer cells. Stromal cells around the tumor showed faint staining; **D**. Benign breast epithelium with MMP-9^−^ staining; **E**. Lymph node without metastases showing MMP-9^−^ staining; **F**. Lymph node with metastases demonstrating MMP-9^++^ staining. Asterisks indicate tumor cells. Magnification, 200×.

MMP-9 protein was localized to the cytoplasm of both tumor and stromal cells (Figure 3, Table 1), as described previously[5].We correlated MMP-9 expression with clinicopathological characteristics, including histological type, tumor size, histological grade, lymph node metastasis and ER, PR and HER2/neu status (Table 2). MMP-9 expression in matched epithelial tissues and lymph nodes was associated with lymph node metastasis (Mann–Whitney U, *P*<0.05). However, protein expression was not correlated with tumor size, histological type, status of ER/PR and HER2, or histological grade (*P*>0.05).

### MMP-9 protein expression and LMVD

LMVD in MMP-9^+^ lymph nodes was significantly higher than in MMP-9^−^ lymph nodes (Mann–Whitney test, *P* 0.001; Table 3). Moreover, MMP-9 staining intensity positively correlated with LMVD in lymph nodes (Spearman's correlation coefficient: 0.569; *P*<0.05).

**Table 3:** Lymphatic microvessel density (LMVD) in the tissue with negative/positive MMP-9 expression.

Tissue type	MMP-9 staining†		U	*P*
	−	+		
Cancer tissues	11.33±11.33 (6)	13.23±11.21 (35)	88.500	0.552
Benign epithelium	1.75±1.50 (20)	2.30±1.10 (21)	50.500	0.315
Lymph nodes	3.43±6.01 (28)	9.92±8.25 (13)	62.000	0.000*

“+”, “++” and “+++” in table 1 for MMP-9 immunochemistry staining were grouped together as “+”.

*P<0.05 is considered statistically significant.

† Values in brackets were number of eligible cases.

## Discussion

MMP-9 is a proteolytic enzyme that degrades basilar membrane and the extracellular matrix. It reportedly promotes cancer progression by increasing cancer cell proliferation, migration, invasion, metastasis and angiogenesis. MMP-9 exert these effects by cleaving a diverse group of substrates, including structural components of the extracellular matrix, growth factor binding proteins, growth factor precursors, receptor tyrosine kinases, cell-adhesion molecules and other proteinases[18].However, reports conflict as to whether increased MMP-9 expression correlates with metastasis and malignancy factors[7].MMP-9 protein is primarily expressed in the cytoplasm of both tumor and stromal cells[5].In this study, its expression in matched epithelium and lymph node tissue was associated with lymph node metastasis. Stromal fibroblasts have been suggested to secrete MMP-9, which is stored and activated in tumor cells[5].Hence, evaluation of stromal MMP-9 expression may provide valuable information on breast cancer prognosis, especially in early carcinogenesis.

A previous study showed that higher expression of MMP-9 protein was associated with lymph node metastasis[19],consistent with our findings. In our study, MMP-9 expression was significantly associated with LMVD and lymph node metastasis. Recent studies have demonstrated that lymphatic networks within lymph nodes expand prior to the onset of metastasis[20].The LMVD reflects the status of lymphangiogenesis and lymphatic vessel remodeling, and when increased, improves opportunities for tumor cells to disseminate to the lymphatic system. Furthermore, it is correlated with lymphangiogenic factors, lymphatic metastasis and poor prognosis in breast cancer[3],as confirmed by our experiments. We found that MMP-9 expression was only associated with lymph node metastasis, but not with other clinical characteristics (Table 2). This implies that MMP-9 plays a major role in the lymphatic system. Thus, as a proteolytic enzyme, MMP-9 may affect the early progression of lymphangiogenesis and lymphatic metastasis of breast cancer.

## Conclusions

We identified higher MMP-9 expression in breast cancer tissue than in benign peritumoral breast epithelium. MMP-9 expression in matched epithelium and lymph nodes was associated with lymph node metastasis, and its expression in lymph nodes was positively associated with lymphangiogenesis. Thus, MMP-9 is a potential clinical marker for breast cancer progression and metastasis.
